# Space Impacts Temporal Processing via a Visual-Dependent Spatially Organized Neural Architecture

**DOI:** 10.1523/JNEUROSCI.1444-24.2025

**Published:** 2025-10-08

**Authors:** Maria Bianca Amadeo, Cristiano Cuppini, Alessia Tonelli, Carolina Tammurello, Walter Setti, Claudio Campus, Sabrina Signorini, Elena Cocchi, Margherita Bonino, Francesca Tinelli, Paola Camicione, Massimiliano Serafino, Monica Gori

**Affiliations:** ^1^Unit for Visually Impaired People (U-VIP), Istituto Italiano di Tecnologia, Genoa 16152, Italy; ^2^Department of Electrical, Electronic, and Information Engineering “Guglielmo Marconi”, University of Bologna, Bologna 40126, Italy; ^3^School of Psychology, The University of Sydney, Sydney 2006, Australia; ^4^Department of Informatics, Bioengineering, Robotics and Systems Engineering (DIBRIS), Università degli Studi di Genova, Genoa 16145, Italy; ^5^Developmental Neurophthalmology Unit, IRCCS Mondino Foundation, Pavia 27100, Italy; ^6^Fondazione Chiossone, Genova, Italy; ^7^Scientific Institute, IRCCS E. Medea, Lecco 23849, Bosisio Parini, Italy; ^8^Department of Developmental Neuroscience, IRCCS Fondazione Stella Maris, Pisa 56128, Italy; ^9^IRCCS Istituto Giannina Gaslini, Genova 16147, Italy

**Keywords:** auditory–tactile, blindness, neural modeling, spatial perception, temporal perception

## Abstract

Establishing the temporal relationship between stimuli challenges the brain, requiring some tolerance for asynchronies to form coherent representations. Based on the theory of implicit causal inference, we hypothesized that temporal processing of events is influenced by spatial features as stimuli coming from the same spatial location are most likely to derive from a common source and, consequently, implicitly merged in time. As visual experience guides the formation of neural sensory maps, we expected the spatial influence on temporal processing to depend on visual experience. In Experiment 1, 41 sighted children and adults (22 females) judged the temporal order of auditory and tactile stimuli delivered to the same or different hands (somatotopic manipulation), with hands either close or far apart (spatiotopic manipulation). In Experiment 2, sighted individuals (15 females) were compared with 26 early blind children and adults (12 females) during the somatotopic manipulation with hands far apart. Results revealed an improvement of temporal resolution with age in sighted individuals, while blind children performed similarly to adults. Notably, spatial features affected the temporal processing of sighted but not blind people, regardless of age. Sighted participants showed higher temporal tolerance toward asynchronies in the case of somatotopic or spatiotopic congruence. A bioinspired neurocomputational model has been developed to unveil neural mechanisms underlying the interaction between spatial and temporal processing. The model demonstrates that temporal processing is mediated by a spatially organized synaptic architecture, which requires visual experience to develop. Without vision, spatial alignment may not be conceptualized as a prior influencing temporal processing.

## Significance Statement

This study demonstrates that spatial features affect temporal resolution of sighted but not blind children and adults. A neurocomputational model suggests these behavioral results stem from spatially organized synaptic connections that require visual experience to develop. This research advances understanding of sensory processes, highlighting the role of vision in developing temporal processing mechanisms, and has implications for interventions in vision impairment.

## Introduction

Every moment, our brain processes a multitude of sensory information that needs to be integrated, segregated, and ordered in space and time to derive a coherent representation of the environment. To tackle this challenge and achieve behavioral flexibility, the brain relies on the shared or different features of the perceived cues, such as temporal synchrony and spatial coincidence (Calvert et al., 2004; Wallace et al., 2004), which help in determining how likely sensory inputs deriving from different sensory modalities belong to common or independent events (Kording et al., 2007; Rohe and Noppeney, 2015). This operation occurs through an implicit causal inference process in which sensory systems implicitly infer when sensory cues should be integrated versus segregated (Wozny et al., 2010).

Despite spatial and temporal features being important determinants for implicit causal inference, the processing of these two pieces of information does not proceed independently and is interconnected. For instance, research suggests that spatial features can, in some cases, interfere with temporal processing. Temporal resolution overall improves when stimuli are presented in different spatial positions (Spence et al., 2003; Zampini et al., 2003; Keetels and Vroomen, 2005; Kitagawa et al., 2005; Poliakoff et al., 2006; Röder et al., 2013) and far rather than close to each other (Roberts et al., 2003; Gallace and Spence, 2005; Shore et al., 2005; Kuroki et al., 2010). In addition, temporal resolution is affected by hand posture, i.e., lower ability to segregate stimuli in time when they are delivered from crossed hands compared with uncrossed (Yamamoto and Kitazawa, 2001; Shore et al., 2002; Schicke and Roder, 2006). Interestingly, it has been shown that the crossed-hand effect on temporal processing is significantly reduced by the sight of uncrossed fake hands (Azanon and Soto-Faraco, 2007) and disappears in case of blindness (Röder et al., 2004), suggesting a possible role of visual experience.

Here, we tested a theoretical framework to delve into the principles and development underlying spatial and temporal processing interaction. We hypothesized that temporal processing is influenced by spatial features because, as suggested by implicit causal inference, stimuli coming from the same spatial location are most likely to derive from a common source and, consequently, to be implicitly merged in time. At the neural level, this could be mediated by a spatial alignment of multisensory maps, which informs the brain that sensory inputs derive from a single crossmodal rather than an independent and separate source, thus influencing temporal processing. Furthermore, since visual experience guides the formation of sensory spatial maps in the brain (Pasqualotto and Proulx, 2012), we hypothesized that the spatial alignment of multisensory maps and, consequently, the spatial influence on temporal processing require vision to develop.

In Experiment 1, we asked sighted children and adults to judge the temporal order of auditory and tactile stimuli, whose relative spatial coordinates were manipulated. Audio and tactile stimuli could be delivered from either the same hand or different hands (somatotopic manipulation), and the two hands were either close or far from each other (spatiotopic manipulation). In Experiment 2, we compared sighted individuals with blind people in discriminating the temporal order of stimuli delivered from the same hand or different hands (somatotopic manipulation) with hands placed far from each other. Finally, to organize these results into a theoretical framework that can guide future experiments, we developed a bioinspired plausible neurocomputational model. This model builds on previously implemented neural networks that describe multisensory perception in spatial and temporal domains (Cuppini et al., 2017a,b, 2020; Monti et al., 2023), with the additional aim of suggesting the most likely neural mechanisms underlying the spatial influence on temporal processing.

## Materials and Methods

### Participants

Sighted and early blind individuals took part in the study. Sighted children were recruited from local schools (Genova, Italy), and sighted adults were recruited from the internal database of the Italian Institute of Technology. All sighted participants reported normal or corrected-to-normal vision and no history of neurological, cognitive, or other sensory–motor deficits.

All blind children were born with visual impairment, while blind adults were classified as early blind if sight loss happened before the age of 6 years old. Blindness was defined as a best-corrected visual acuity worse than 1.3 LogMAR according to the criteria established by the World Health Organization. All blind participants reported normal hearing and no history of neurological, cognitive, or other sensory–motor deficits except for total blindness. Blind children were recruited from six rehabilitation centers in Italy. Blind adults were recruited from the internal database of the Italian Institute of Technology.

All participants were native Italian speakers. The local health service ethics committees approved the study (multicentric protocol approved by the Ethics Committee of ASL 3 Genova, Committee of the Meyer's Hospital, The Ethics Committee of Pavia Area, Fondazione IRCCS Policlinico San Matteo Pavia, and the Ethics Committee of IRCCS Eugenio Medea, sezione Scientifica dell’Associazione “La nostra Famiglia”: Prot. IIT_UVIP_MySpace N. 268/2021, May 3, 2021). The research protocol was conducted in line with the Declaration of Helsinki. Adults provided written informed consent prior to testing, and written informed parental consent was obtained from all the children**.**

#### Experiment 1

For Experiment 1, 27 sighted children and 16 sighted adults were recruited. One sighted child and one sighted adult have been excluded from the analyses because they were identified as outliers [i.e., their just noticeable difference (JND) differed more than three standard deviations from the group's mean score]. Thus, the remaining participants for Experiment 1 comprised 26 sighted children (mean ± SD = 11.5 ± 3.5 years old; female, 13) and 15 sighted adults (25.7 ± 5.9 years old; female, 9).

#### Experiment 2

For Experiment 2, 28 blind individuals aged between 6 and 55 years old were recruited and compared with age-matched sighted subjects. One blind child and one blind adult were identified as outliers and excluded. The final sample for blind people consisted of 16 blind children and 10 blind adults. To reach age- and gender-matched samples, some sighted participants (*N* = 18) were extracted from the sighted sample of Experiment 1, and some others (*N* = 8) were tested for the first time only with Experiment 2. The final sample for sighted people consisted of 16 sighted children and 10 sighted adults. Specifically, sighted and blind children were matched on a one-to-one basis for age (sighted children, 11.9 ± 3.5 years old; female, 10; blind children, 11.8 ± 3.4 years old; female, 6). Sighted and blind adults were not statistically different in terms of age (sighted adults, 33.7 ± 7.6 years old; female, 5; blind adults, 41.1 ± 14.3 years old; female, 6; comparison for age, *t*_(13.8)_ = 1.4; *p* = 0.2).

### Apparatus and stimuli

**Figure 1.**
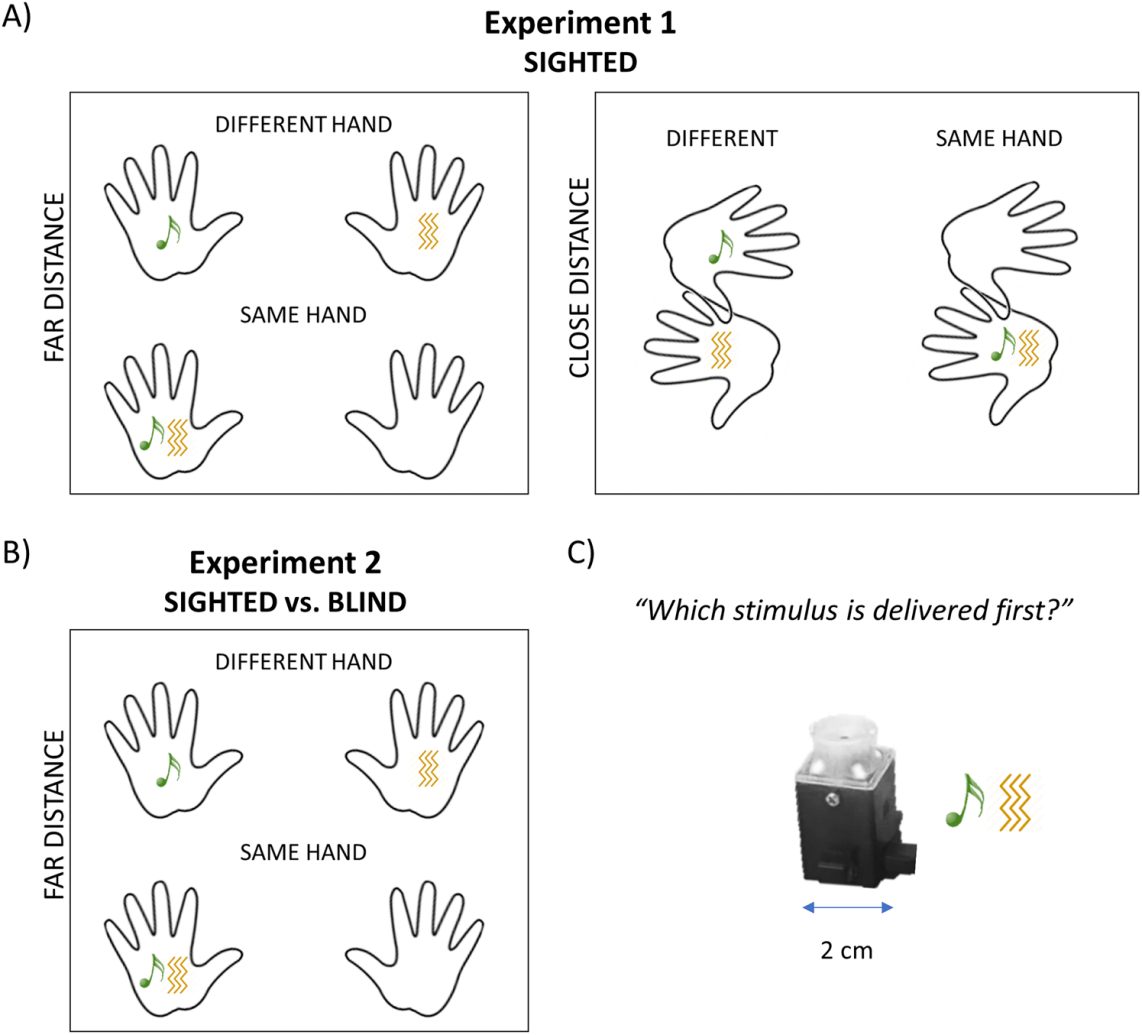
Graphical representation of experimental paradigm and setup. Participants were presented with pairs of audiotactile stimuli separated by various onset asynchronies and asked to judge which stimulus (i.e., auditory or tactile) appeared first. ***A***, Experiment 1, audiotactile stimuli could be delivered from either the same hand or different hands; the hands could be either close or far from each other. ***B***, Experiment 2, audiotactile stimuli could be delivered from either the same hand or different hands, but hands were placed far from each other. ***C***, The device is placed on the back of the hand to reproduce the auditory and tactile stimulation.

The auditory and vibrotactile stimuli were delivered by two custom-built serial–controlled audiotactile stimulators (Gori et al., 2019) fixed on the back of the hands through a transparent film, which did not impact the stimulations (Fig. 1*C*). Both stimuli were delivered from the same device. The vibrotactile stimulus was a 10 ms continuous pure vibration (230 Hz). The auditory stimulus was a 10 ms 1,000 Hz pure tone. Both the auditory and tactile stimuli were generated and temporally controlled by MATLAB (MathWorks) with the Psychtoolbox-3 package (Brainard, 1997; Pelli, 1997; Kleiner et al., 2007). All timings were verified with an oscilloscope prior to testing.

### Design

In both Experiment 1 and Experiment 2, participants were blindfolded and performed a temporal-order judgment task. They were presented with pairs of audiotactile stimuli with different stimulus onset asynchronies (SOAs) and were instructed to report which stimulus appeared first. Negative SOAs represent auditory-leading trials and positive SOAs represent tactile-leading ones. The SOA was determined adaptively by a QUEST routine on a trial-by-trial basis. QUEST is an adaptative algorithm that uses a Bayesian approach to set the SOA using all the information available from previous trials, supplemented by prior knowledge from the literature (Watson and Pelli, 1983; Watson, 2017). The SOA ranged from 0 to 800 ms. The maximum SOA was set to ±800 ms based on previous studies exploring similar audiotactile mechanisms in blindness and children (Occelli et al., 2008; Stanley et al., 2019).

Beyond stimulus temporal onset, the hand from where the stimuli were delivered and hand positions were modulated to explore the role of spatial features on auditory–tactile temporal discrimination skills.

#### Experiment 1

In Experiment 1, we manipulated both somatotopic and spatiotopic spatial coordinates as audio and tactile stimuli could be delivered from either the same hand or different hands (somatotopic manipulation), and the two hands were either close or far from each other (spatiotopic manipulation). See Figure 1*A* for a graphical representation. Participants performed two blocks in a randomized order. In one block, participants’ hands were positioned on the table far away from each other (i.e., “far distance”). Hands were aligned with the shoulders, ∼40 and 60 cm away from the body for children and adults, respectively. In the other block, the hands of participants were positioned on the table in front of the body midline (i.e., “close distance”). To reach the final hand position, participants were instructed to place their hands on the table aligned with the shoulder and, from this position, move their forearms to reach the body midline with their hands. Half of the participants performed the experiment with the right hand in front of the left hand, and half of the participants did the opposite (i.e., the left hand in front of the right hand). Within each block, auditory and tactile stimuli could be delivered from either the same hand or from two different hands. This gave rise to two levels of hand congruence, namely, (1) “same hand”, auditory and tactile stimuli from the right hand or auditory and tactile stimuli from the left hand, and (2) “different hand”, auditory stimulus from the right hand and tactile stimulus from the left hand or auditory stimulus from the left hand and tactile stimulus from the right hand. Trials from “same hand” and “different hand” were randomized within a block. In sum, adults performed 120 trials for “far distance” (i.e., 60 “same hand” and 60 “different hand”) and 120 trials for “close distance” (i.e., 60 “same hand” and 60 “different hand”), resulting in 240 trials. Children performed 60 trials for “far distance” (i.e., 30 “same hand” and 30 “different hand”) and 60 trials for “close distance” (i.e., 30 “same hand” and 30 “different hand”), resulting in 120 trials in total.

#### Experiment 2

In Experiment 2, only the block “far distance” of Experiment 1 was performed (Fig. 1*B*). As such, blind and age-matched sighted participants were tested with the hands placed on the table far from each other, aligned with the shoulders, ∼40 and 60 cm away from their body for children and adults, respectively. As for Experiment 1, the auditory and tactile stimuli could be delivered from either the same hand or different hands. Trials from the “same hand” and “different hand” were randomized. Adults performed 120 trials (i.e., 60 “same” hand and 60 “different” hand), while children performed 60 trials (i.e., 30 “same” hand and 30 “different” hand).

### Experimental procedure

The participant sat at a desk with both hands on the table and the audiotactile stimulators on the back of them (one for each hand). The experimenter sat at the computer keyboard to the participant's right side. All participants were blindfolded (i.e., their hands were not visible) and wore over-ear headphones throughout the experiment to mask any noise emitted by vibrotactile stimulators (this setup was tested in a pilot study). They were instructed to maintain a stable head position. However, the researcher continuously monitored head and body orientation during the experiment. Responses were given orally, and the experimenter keyed the responses into the computer manually and initiated the next trial. The maximum response time was fixed to 10 s for trial; after that, the next trial occurred.

To ensure children understood the instructions correctly, a short training session with feedback was conducted before starting. The experimental protocol began once we were sure they understood the task. No feedback was given during experimental sessions. Participants were encouraged to take a break between blocks. Not including breaks, each experiment took ∼6 and 12 min for children and adults, respectively.

### Statistical analyses

All analyses were conducted using R (R Core Team, 2013). First of all, for each participant and condition of Experiment 1 (i.e., “same hand–far distance”, “different hand–far distance”, “same hand close distance”, “different hand–close distance”) and Experiment 2 (i.e., “same hand”, “different hand”), the proportion of trials where the tactile stimulus was perceived as occurring before the auditory stimulus was plotted as a function of SOA value and fitted with a cumulative Gaussian function. Following standard psychophysical procedure, the best fitting function's mean and standard deviation were obtained as estimates of point of subjective equality (PSE) and JND. The PSE represents the perceptual bias of a participant’s perception of auditory–tactile synchrony and indicates the participant’s accuracy, while the JND represents the smallest temporal difference between auditory and tactile signals that an individual could detect and is an index of precision. For some participants (*N* = 1 in Experiment 1; *N* = 3 in Experiment 2), a JND of 0 ms was assigned due to error-free performance. This reveals a limitation in our design, which could not detect temporal intervals smaller than 23 ms.

#### Experiment 1

Statistical analyses were performed on the group of sighted individuals to explore the developmental trend of the audiotactile temporal discrimination skills and the impact of spatial cues on it. Statistical analyses aimed at exploring the impact of spatial coordinates of audiotactile stimuli on the temporal binding skills of sighted participants while controlling for the effect of age. We fitted linear mixed-effect models on data to examine the effects and interactions of hand (“same,” “different”), distance (“far,” “close”), and age on JND and PSE. Model fitting was done using the *lmer* function of the *lme4* package (Bates et al., 2014), while the significance of effects was assessed using the *Anova* function of the *car* package (Fox and Weisberg, 2018), providing not an ANOVA but an analysis of deviance table (Type III Wald *χ*^2^ tests). Significant fixed effects were further investigated with follow-up linear mixed-effect models and the *emmeans* function of the *emmeans* package (Lenth et al., 2020) by obtaining estimated marginal means (EMMs) and computing their contrasts. *P* values from the follow-up analyses were corrected using the Bonferroni’s method for multiple comparisons.

According to Wilkinson's notation (Wilkinson and Rogers, 1973), the models fitted were as follows:

1. JND ∼ Hand (*Same*, *Different*) × Distance (*Far*, *Close*) × Age + (1|subject)
2. PSE ∼ Hand (*Same*, *Different*) × Distance (*Far*, *Close*) × Age + (1|subject)

Hand, distance, and age were included as fixed effects, while subject was treated as a random effect. This approach allows us to model individual differences while still assessing the overall effects of hand, distance, and age on JND. For completeness, the model for JND was also fitted to the data excluding participants with JND = 0. A segmented regression analysis was performed to identify possible breakpoints to deepen the effect of age. Results of PSE are reported in Text S2 (Table S2) in Supplementary Materials.

#### Experiment 2

Statistical analyses aimed at exploring differences between sighted and blind groups related to the effect of spatial cues on temporal processing. Using a similar approach to Experiment 1, the fitted models were as follows:

1. JND ∼ Hand (*Same*, *Different*) × Group (*Sighted*, *Blind*) × Age + (1|subject)
2. PSE ∼ Hand (*Same*, *Different*) × Group (*Sighted*, *Blind*) × Age + (1|subject)

Hand, group, and age were included as fixed effects, while subject was treated as a random effect. This approach allows us to model individual differences while still assessing the overall effects of hand, group, and age on JND. Significant fixed effects were further investigated with follow-up linear mixed-effect models and the *emmeans* function of the *emmeans* package (Lenth et al., 2020) by obtaining EMMs and computing their contrasts. *P* values from the follow-up analyses were corrected using the Bonferroni’s method for multiple comparisons. For completeness, the model for JND was also fitted to the data excluding participants with JND = 0.

Since a significant interaction between group and age emerged when predicting JND values, we statistically tested for slope differences between sighted and blind groups with *emtrends* function of *emmeans* package (Lenth et al., 2020). Results of PSE are reported in Text S2 (Table S3) in Supplementary Materials.

### Model architecture

To explore the mechanisms likely involved in the interaction between spatial constraints and temporal processing in the case of crossmodal stimulations, we implemented and tested a physiologically plausible neurocomputational model based on previous neural architectures realized to study unisensory and multisensory processing (Cuppini et al., 2017a,b, 2020; Crosse et al., 2022; Monti et al., 2023). In these computational efforts, we identified several neural and architectural elements responsible for processing spatial and temporal features of external stimuli. In the spatial domain, (1) the topological organization among elements of the simulated sensory cortices and the reciprocal intercortical connectivity are fundamental to reproducing the crossmodal influence on the spatial perception of external events and underlie the ability to solve the causal inference problem (Cuppini et al., 2017a). In the temporal domain, (2) the direct reciprocal excitatory projections, characterized by a fast temporal dynamic, have a direct effect on the temporal window of integration (Spence and Squire, 2003; Diederich and Colonius, 2009; Vroomen and Keetels, 2010), while (3) the inhibitory feedbacks among primary unisensory areas, characterized by a slow temporal dynamic, are responsible for the modality switch-cost effect (Cuppini et al., 2020; Monti et al., 2023). The present model kept all the above mechanisms (i.e., input and interneurons layers) as in previous experiments. The output layer, instead, is task-specific, and it has been added specifically to study and simulate how the temporal and spatial features of auditory and tactile stimuli are interconnected and affect the JNDs of the participants, mimicking results of Experiment 2.

**Figure 2.**
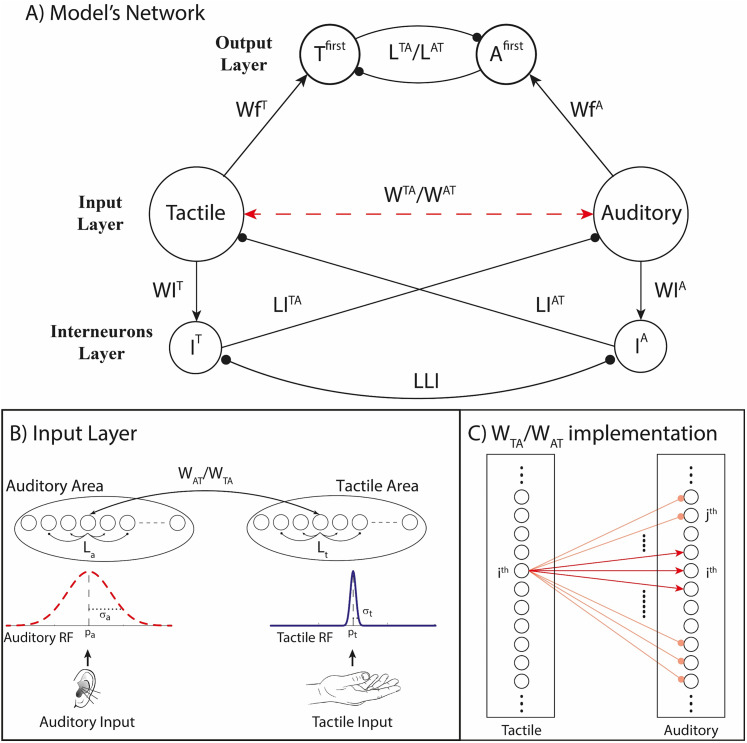
Model’s architecture. ***A***, The network presents three layers. The input regions, tactile and auditory, receive the external inputs. They are reciprocally connected through crossmodal synapses (W^TA^ and W^AT^) and send excitatory connections to the interneurons populations (WI^T^ and WI^A^), which mediate a long-term competition among the different sensory modalities and to the output elements (Wf^T^ and Wf^A^). Interneurons (I^T^ and I^A^) send inhibitory feedback projections to the input regions of the other sensory modality (LI^TA^ and LI^AT^) and are mutually inhibited by means of a WTA mechanism mediated by the synapses LLI. The output elements (T^first^ and A^first^) exchange reciprocal inhibition through L^TA^ and L^AT^ synapses, implementing a competition. ***B***, Synaptic implementation in the input layer and external stimuli. The input regions are topologically organized through the intra-area synapses (L), which excite nearby elements and inhibit distant ones. The external inputs are implemented by means of Gaussian functions, simulating the different RFs of the two sensory modalities. ***C***, Synaptic architecture of the crossmodal synapses. Elements spatially aligned are reciprocally excited (red arrows), while elements sensitive to distant portions of the space exchange mutual inhibition (orange lines).

The network presents three layers (Fig. 2). The first (1) is the input layer, which consists of auditory and tactile, mimicking the primary sensory cortices that receive the external stimuli. These two regions are made of two arrays of 180 neural elements, reciprocally connected by means of the synapses W^AT^ and W^TA^. Elements in the tactile area follow a somatotopic arrangement, while auditory elements are topographically organized. Input regions send excitatory projections to the corresponding arrays of inhibitory interneurons, WI^T^ and WI^A^, and the two neural elements in the output layer, Wf^T^ and Wf^A^.

The second layer (2) is the interneurons layer, which consists of modality-specific inhibitory interneurons (I^T^ and I^A^) competing one another through a winner-takes-all (WTA) mechanism, mediated by inhibitory synapses LLI. This layer implements a long-lasting cross–sensory competition (mediated by the inhibitory feedback projections LI^TA^ and LI^AT^) between the two sensory modalities when stimuli are presented sequentially. The processing of the current stimulus is thus affected by the previous one, when they are of the opposite sensory modality, and the dynamics of this mechanism are slow.

Finally, (3) the output layer consists of two modality-specific neural elements (T^first^ and A^first^) reciprocally interconnected by inhibitory synapses, L^AT^ and L^TA^, and stimulated by long-range excitatory synapses from neural elements of the corresponding input region (Wf^T^ and Wf^A^). This layer is responsible for the temporal judgment of the stimuli presented to the network, simulating neural assemblies in the prefrontal regions sensitive to the temporal-order presentation of external inputs (Grossberg, 2013).

As mentioned above, parameters of the unisensory and interneurons layers have been kept the same as in previous papers (Cuppini et al., 2017a,b, 2020; Crosse et al., 2022; Monti et al., 2023), only adapting those needed to mimic the population's results for this specific task. Details about the mathematical implementation of the network can be found in Text S1 and Table S1 in Supplementary Materials (Treves, 1993; Ben-Yishai et al., 1995; Jansen and Rit, 1995; Recanzone, 1998; Wendling et al., 2002; Kayser et al., 2005; Cuppini et al., 2014, 2020; Zhang et al., 2020; Fu and Riecke, 2023).

### Assessment of network performance

The model structure was tested stimulating the network with auditory and tactile inputs in different spatial (i.e., same hand and different hands) and temporal (i.e., SOAs) configurations, with the onset and duration chosen to mimic the experimental setup described above for Experiment 2. Data of Experiment 2 have been compared with the model results. The presentation onset of the stimuli was systematically modified to identify the minimum temporal disparity (i.e., JND) necessary for the network to temporally discriminate the order of the two stimuli. Specifically, external stimuli were excitatory inputs with an assigned efficacy, $I_{0}^{A}\;\;\mathrm{and}\;\;I_{0}^{AT}$, and a duration *D^r^* = 10 ms, presented with an SOA ranging from 0 to 300 ms.

The “output layer” mimics the ability of simulated subjects to correctly identify the order of the presented stimuli of the two modalities. Two elements implement this layer, A^first^ and T^first^ (Fig. 2), each of them sensitive to one of the two sensory modalities. When the corresponding sensory input stimulates the corresponding input area, the elicited activity generates an excitatory input to the output element, generating an activity. The first element activated would signal the stimulus perceived as the first one presented, but only if it is the only active element in the output layer. If both elements would be stimulated above a detection threshold, *φ* (10% of the maximum neurons' activity), then the model is unable to discriminate the temporal order of the presented stimuli. To achieve the correct detection, this network implements a cross-sensory competition between the elements coding for the temporal feature of two sensory modalities when stimuli are presented sequentially: the first activated inhibits the element coding for the second sensory modality.

## Results

### Experiment 1

**Figure 3.**
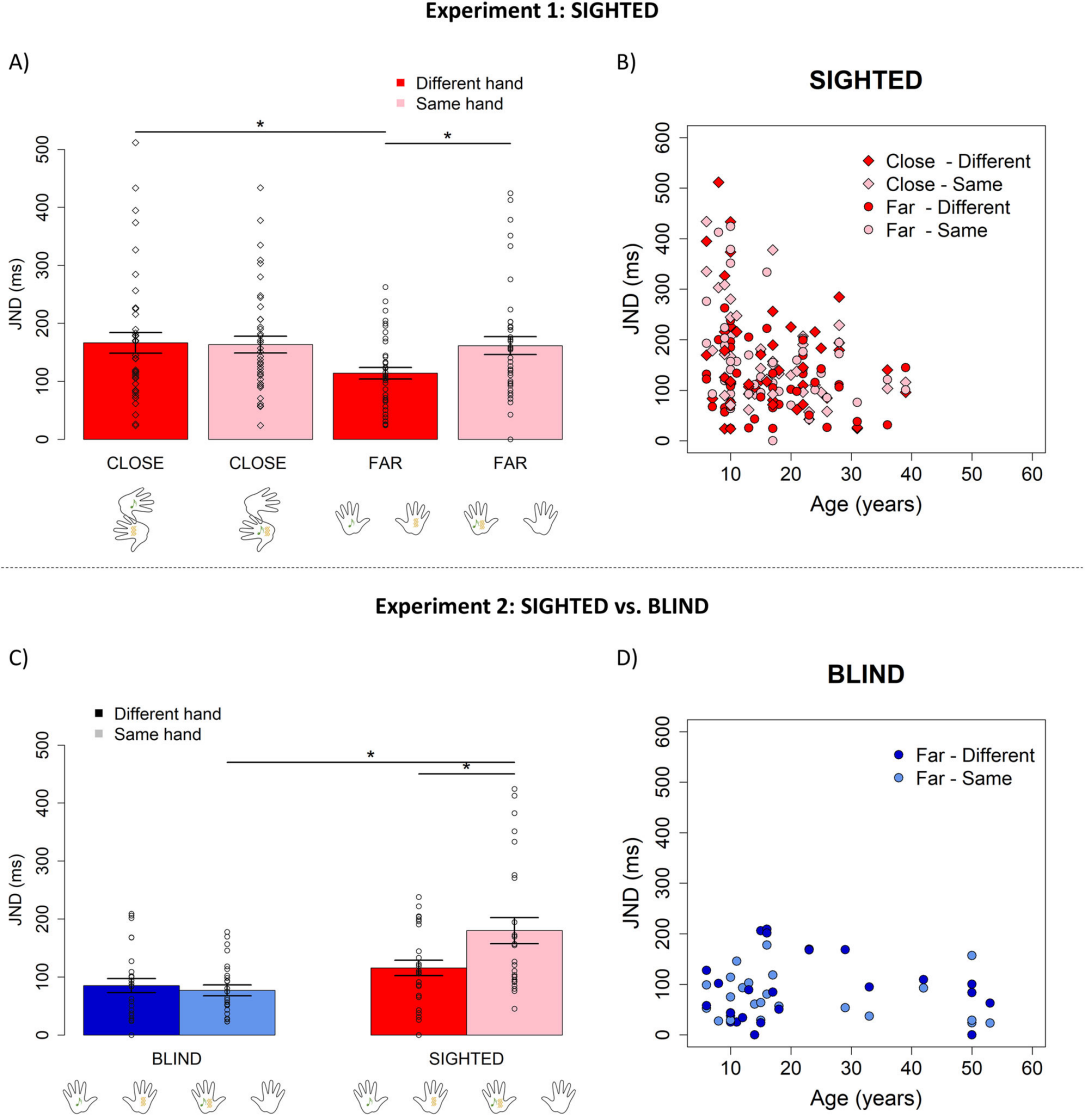
Results of Experiments 1 and 2. In Experiment 1 (top panel), hands were placed either close or far away from each other, and stimuli were delivered from either the same hand or different hands. ***A***, JNDs (mean ± standard deviation) for sighted participants for each condition; points represent single participants. ***B***, JNDs plotted as a function of age for sighted participants. In Experiment 2 (bottom panel), sighted and blind participants were compared when stimuli were delivered from either the same hand or different hands with hands placed far from each other. ***C***, JNDs (mean ± standard deviation) of sighted (red) and blind (blue) participants; points represent single participants. ***D***, JNDs plotted as a function of age for blind participants.

To disentangle the effect of spatial proximity and coincidence on temporal tolerance to asynchronies, in Experiment 1, we tested 26 sighted children and 15 sighted adults while changing somatotopic (i.e., same hand vs different hands) and spatiotopic (close vs far distance between the hands) spatial coordinates of audiotactile stimuli (Fig. 1*A*). Results of the linear mixed model with individual JNDs as a dependent variable and age, hand (“same,” “different”), and distance (“close,” “far”) as predictors (Table 1) revealed a significant effect of age ( $\chi_{\left(1\right)}^{2}=7.14$; *p* = 0.007), hand ( $\chi_{\left(1\right)}^{2}=4.89$; *p* = 0.03), and distance ( $\chi_{\left(1\right)}^{2}=7.23$; *p* = 0.007) and a significant interaction between hand and distance ( $\chi_{\left(1\right)}^{2}=6.28$; *p* = 0.01). Post hoc analyses on the interaction revealed that participants’ temporal resolution in judging the order of sensory stimuli was better when the stimuli came from different hands and far spatial locations than when they were delivered to the same hand or close hands (Fig. 3*A*; Table 2). Importantly, the significant interaction between age, hand, and distance held when analyzing the data excluding one participant with JND = 0 ( $\chi_{\left(1\right)}^{2}=6.61$; *p* = 0.01).

**Table 1.** Results of the model JND ∼ Hand(same, different) × distance (far, close) × age + (1|subject)

Predictors	*χ*^2^ (df = 1)	*p*
Hand	4.89	0.03
Distance	7.23	0.007
Age	7.14	0.007
Hand × age	1.65	0.2
Distance × age	1.13	0.3
Hand × distance	6.28	0.01
Hand × distance × age	1.01	0.3

Underlined values indicate statistically significance of results (*p* < 0.05).

**Table 2.** Results of EMMs addressing the interaction between hand (same, different) and distance (close, far) in sighted participants

Contrasts		Estimate	Standard error of the mean (SEM)	*T* ratio (df = 117)	*p*
Close distance same hand	Close distance different hand	2.95	14.2	0.21	0.9
Far distance same hand	Far distance different hand	−47.4	14.2	−3.34	0.002
Close distance same hand	Far distance same hand	1.84	14.2	0.13	0.9
Close distance different hand	Far distance different hand	52.2	14.2	3.67	0.0008

Underlined values indicate statistically significance of results (*p* < 0.05).

As for age (Fig. 3*B*), a subsequent segmented regression analysis on age identified a breakpoint at 13 years of age (standard error, ±2.5 years old; 95% confidence intervals, [8.08–17.92 years old]). See Supplementary Materials for results of PSE.

### Experiment 2

To investigate the role of vision on the influence of spatial constraints on temporal processing, in Experiment 2 we compared the temporal precision of 16 early blind children and 10 early blind adults with the temporal precision of age-matched sighted individuals in discriminating the temporal order of stimuli under somatotopic manipulation (i.e., “same hand” vs “different hand”) with hands placed far from each other. The linear mixed model with individual JNDs as the dependent variable and age, group (“sighted,” “blind”), and hand (“same”, “different”) as predictors (Table 3) yielded noteworthy results (see also Supplementary Materials for results of PSE). A significant interaction emerged between group and age ( $\chi_{\left(1\right)}^{2}=11$; *p* = 0.0009), indicating a different relationship between age and JNDs across the sighted and blind groups. Post hoc analyses showed that age emerged as a significant predictor of temporal precision for the sighted group ( $\chi_{\left(1\right)}^{2}=13.23$; *p* = 0.0002), while it did not significantly predict JNDs among blind individuals ( $\chi_{\left(1\right)}^{2}=0.1$; *p* = 0.7; Fig. 3*D*). This was also confirmed by subsequent comparisons highlighting a significant difference (*p* = 0.0017) in the trend of age's influence on JND between the blind (estimated trend ± standard error = −0.20 ± 0.7) and sighted (−4.06 ± 0.9) groups. Another significant interaction was observed between the group and hand ( $\chi_{\left(1\right)}^{2}=10.74$; *p* = 0.001), suggesting distinct relationships between somatotopic manipulation and JND in sighted versus blind individuals regardless of age (Fig. 3*C*). Post hoc analyses (Table 4) revealed that the precision of sighted participants varied when the stimuli were delivered from the same hand or different hands as noticed in Experiment 1. Instead, the temporal processing of blind people was not significantly different between the two conditions. Moreover, blind and sighted participants performed similarly when the stimuli came from different hands, but blind individuals outperformed sighted ones when stimuli were delivered from the same hand. Importantly, the significant interactions between group and age ( $\chi_{\left(1\right)}^{2}=9.24$; *p* = 0.002) and between group and hand ( $\chi_{\left(1\right)}^{2}=11.96$; *p* = 0.008) held when analyzing the data excluding participants with JND = 0.

**Table 3.** Results of the model JND ∼ Hand (same, different) × group (sighted, blind) × age + (1|subject)

Predictors	*χ*^2^ (df = 1)	*p*
Hand	7.3	0.007
Group	15.23	0.00009
Age	7.3	0.007
Hand × age	4.2	0.04
Hand × group	10.74	0.001
Age × group	10.99	0.009
Hand × group × age	3.18	0.07

Underlined values indicate statistically significance of results (*p* < 0.05).

**Table 4.** Results of EMMs addressing the interaction between hand (same, different) and group (sighted, blind)

Contrasts		Estimate	Standard error of the mean	Degrees of freedom	*T* ratio	*p*
Sighted group same hand	Sighted group different hand	−59.83	14.8	48	−4	0.004
Blind group same hand	Blind group different hand	7.39	14.8	48	0.5	0.9
Sighted group same hand	Blind group same hand	−94.4	19.2	82.5	−4.9	<0.0001
Sighted group different hand	Blind group different hand	−27.2	19.2	82.5	−1.4	0.4

Underlined values indicate statistically significance of results (p < 0.05).

### Model’s simulations

To explore the mechanisms likely involved in the interaction between spatial constraints and temporal processing in the case of crossmodal stimulations, we mimicked the experimental conditions of Experiment 2: the model has been used to mimic the behaviors of 26 simulated blind participants and 26 simulated sighted participants, when the stimuli were delivered from either the same hand or different hands, with hands placed far from each other.

**Figure 4.**
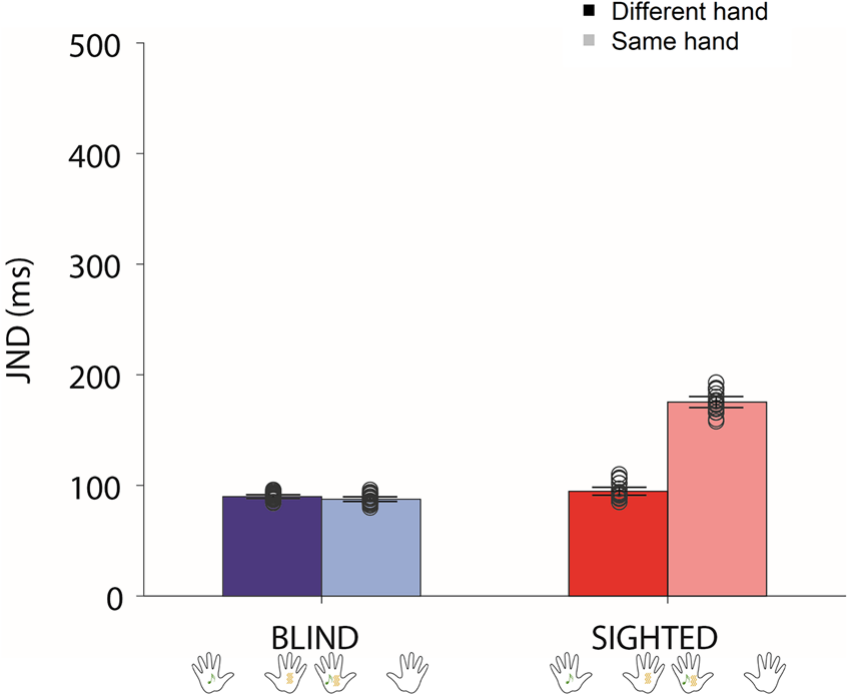
Results of simulations. We mimicked the experimental conditions of Experiment 2: JNDs (mean ± standard deviation) of 26 simulated blind (blue) participants and 26 simulated sighted (red) participants were compared when stimuli were delivered from either the same hand or different hands with hands placed far from each other; points represent single participants.

In the case of stimuli of different modalities presented to the network, both output neural elements receive an excitatory stimulus. If the reciprocal competition leaves active only one of them, the model perceives the corresponding sensory modality as the first one presented in input; if both elements are active instead, the model cannot make a judgment about which one was the first perceived and does not discriminate the temporal order between the two stimuli (i.e., higher temporal tolerance to asynchrony and worse temporal resolution). The results of the simulations are reported in Table 5 and in Figure 4.

**Table 5.** Mean JNDs in case of the same hand or different hands stimulated, evaluated over a population of 26 sighted and a population of 26 blind simulated subjects

Sighted		Blind	
Same hand	Different hands	Same hand	Different hands
175.4 ± 9.7 ms	94.7 ± 7.1 ms	87.6 ± 4.1 ms	89.9 ± 3.3 ms

The results of the simulations confirmed what emerged in Experiment 2 for sighted and blind participants. In sighted participants, stimuli presented to the same hand (spatially congruent) tend to be recognized as synchronous even if presented with a longer asynchrony (i.e., higher JND); vice versa, stimuli presented to the different hands (spatially incongruent) are temporally segregated even for shorter asynchrony (i.e., lower JND).

## Discussion

In this work, we provide compelling evidence that spatial features influence temporal processing, already in childhood but only in the presence of visual experience. Specifically, when stimuli are spatially congruent stimuli, sighted individuals exhibit worse temporal resolution and an increased tendency to bind stimuli in time. A biologically inspired neurocomputational model suggests that these findings result from spatially organized crossmodal connections, which develop only when there is a previous visual experience of spatially aligned audiotactile stimuli.

First of all, we observed that precision in temporal discrimination improves with age, independently of the spatial manipulations, for sighted but not blind individuals. Notably, 13 years of age emerged as a significant developmental breakpoint for sighted children, whereas blind children already performed at the level of both sighted and blind adults. Results for sighted individuals align with previous studies showing that temporal processing gradually fine-tunes between 10 and 13 years of age based on the sensory modalities involved (Chen et al., 2016, 2018; Stanley et al., 2019). Instead, no previous studies investigated the development of audiotactile temporal binding in blind children. The earlier development of blind children compared with sighted ones could be due to the necessity of compensating for the lack of visual information, supporting the idea of crossmodal plasticity (Collignon et al., 2009). This happens, for instance, for optimal audio-haptic integration, which develops earlier during development in blind individuals (Scheller et al., 2021).

Most importantly, Experiment 1 suggests a worse temporal resolution and a higher tendency to bind stimuli in time in case of either somatotopic or spatiotopic congruence for sighted individuals, regardless of age. Other studies previously demonstrated this effect for audiovisual and visuotactile stimuli, showing that spatial cues can affect temporal processing (Spence et al., 2003; Keetels and Vroomen, 2005). Only two studies previously explored audiotactile temporal processing considering the spatial coordinates of stimuli (Kitagawa et al., 2005; Zampini et al., 2005), revealing conflictual results. On the one hand, Zampini et al. (2005) did not find an interaction between space and time. On the other hand, Kitagawa et al. (2005) observed a spatial modulation of audiotactile temporal processing when the stimuli were presented from behind participants’ heads. Similarly to our results, temporal resolution increased when the auditory and tactile stimuli were presented from different spatial positions rather than from the same position. One possibility to reconcile these data is to consider the overall higher temporal resolution observed by Zampini et al. (2005), which raises the possibility of a ceiling effect: participants might already excel to such an extent, possibly due to the reliability of the stimuli employed, that the spatial effect may not be readily evident. Here, we add to previous research that the interaction between spatial cues and temporal processing also exists for audiotactile stimuli in the frontal space, and it is already present in children as young as 6 years of age. Moreover, we pointed out that somatotopic coincidence and spatiotopic proximity have a similar effect. It would have been plausible to hypothesize that somatotopic coincidence is more powerful than spatiotopic proximity in worsening temporal-order discrimination of stimuli. Interestingly, this was not the case, highlighting that the spatial cues affect temporal processing even when spatial positions do not coincide. We speculate this phenomenon occurs because auditory receptive fields (RFs) typically exhibit a broader spatial width than tactile RFs, reflecting the lower spatial precision of auditory spatial cues. This broader tuning would allow for overlap between auditory and tactile RFs without necessitating precise spatial coincidence. We interpret these results within the framework of implicit causal inference (Wozny et al., 2010; Odegaard et al., 2015). When presented with sensory stimuli, the brain faces the challenges of implicitly understanding the causal structure within the signals and the source(s) that generated them. Experiment 1 shows that, in addition to the actual physical temporal discrepancy between the audiotactile signals, the perceptual experience of synchrony depends on the spatial constraints.

Notably, Experiment 2 revealed that the spatial manipulation did not affect the temporal processing of blind people. Indeed, temporal precision of blind participants was similar when segregating stimuli delivered from the same hand or different hands, suggesting that spatial coincidence does not impact their temporal binding tendency. This lack of spatial influence agrees with previous studies showing the role of vision and sensory experience on the cross-hand effect on temporal processing (Röder et al., 2004; Azanon and Soto-Faraco, 2007; Badde et al., 2019; Maij et al., 2020; Hong et al., 2022). It has been observed that the sight of hand distance also modulates tactile temporal-order judgments, with the worse temporal resolution when the hands were perceived as being close rather than further apart due to the mirror reflection (Gallace and Spence, 2005). Since blind and sighted participants showed a similar performance when stimuli were delivered from different positions, we exclude that our findings are driven by the overall superior temporal skills of blind people. This is also evident in other studies showing similar temporal processing between sighted and blind adults when tactile stimuli were delivered from uncrossed hands (Crollen et al., 2017, 2019). Since the different hand condition is performed similarly between blind and sighted individuals, one possibility is also that the superior tactile representation of blind people (Goldreich and Kanics, 2006; Alary et al., 2009; Voss, 2011) affects the cross-modality judgment in the same hand condition. The only study exploring audiotactile temporal processing in blind adults examined the relative spatial position of stimuli (Occelli et al., 2008), uncovering a somatotopic modulation among blind participants akin to our observation in sighted individuals. However, the latter outcome might be influenced by the heterogeneity of their blind sample, encompassing individuals born with visual impairments (sample size, *N* = 8) and those who acquired blindness later in life (sample size, *N* = 9). Since late blind individuals often perform similarly to sighted individuals due to visual experience early in life, a sample of early blind participants, as in this study, may be necessary to highlight the role of vision in developing the interaction between spatial features and temporal tolerance to asynchronies. Alternatively, late blind individuals may have less developed tactile processing abilities compared with early blind individuals, making them more influenced by the somatotopic domain. Interestingly, an additional factor that future studies could examine is the role of Braille knowledge in temporal processing.

The neurocomputation model we developed suggests that the spatial influence observed in sighted but not in blind individuals is mediated by the synaptic organization of crossmodal connections between elements whose RFs are spatially overlapping in the two input regions. The reciprocal interaction among these elements adds an excitatory component that could offset the temporal asynchrony between the two stimuli, generating a likely perception of synchronicity. When the stimuli are placed on different hands, the reciprocal synapses are inhibitory, which favors the ability of the brain to keep segregated in time the two percepts: the first presented in time would inhibit the ability of the following one to stimulate the corresponding sensory region as fast as it would occur without such crossmodal competition. This finding supports the hypothesis that a spatial alignment of sensory maps for audio and tactile modalities influences the temporal domain, causing the system to integrate instead of segregate asynchronous stimuli, leading to worse temporal resolution. Results of blind individuals, instead, are explained in the model by the lack of excitatory connections among spatially aligned input regions. The lack of visual experience with spatially congruent audiotactile pairs would prevent the development of such organized excitatory connectivity, which results in the absence of additional excitation in case of stimuli delivered from the same hand.

Overall, the model's simulations support that spatially aligned maps directly affect temporal processing and the ability to integrate versus segregate the stimuli in time. This alignment in the model is mediated by crossmodal connections topographically organized so that sensory neural elements sensitive to the same spatial locations tend to excite reciprocally, while elements whose spatial RFs are spatially misaligned inhibit one another. In blind individuals, such spatially organized synaptic architecture may be lacking, and this could explain why the spatial relationship among sensory cues does not influence the temporal-order judgment.

To conclude, our behavioral findings demonstrated an interaction between spatial features of stimuli and their temporal processing, regardless of age, but only in the case of visual experience. Specifically, sighted individuals showed higher temporal tolerance toward audiotactile asynchronies in the case of somatotopic coincidence and spatiotopic proximity, which means a higher tendency to bind events in time in the case of spatial congruence. Based on the model’s simulation, we speculate that in the case of spatial congruence, auditory and tactile spatial representations of sighted people overlap, giving rise to a prior that implicitly influences temporal binding. Instead, auditory and tactile spatial representations may not be spatially aligned without vision, resulting in spatially independent unisensory representations. This would explain why the temporal resolution of blind people is unaffected by spatial features of audiotactile stimuli. Within this framework, spatial congruence may not be conceptualized by blind people as a prior implicitly influencing temporal binding and would, therefore, be irrelevant to temporal processing.
